# The road to molecular identification and detection of fungal grapevine trunk diseases

**DOI:** 10.3389/fpls.2022.960289

**Published:** 2022-08-26

**Authors:** Filipe Azevedo-Nogueira, Cecília Rego, Helena Maria Rodrigues Gonçalves, Ana Margarida Fortes, David Gramaje, Paula Martins-Lopes

**Affiliations:** ^1^DNA & RNA Sensing Lab, School of Life Sciences and Environment, University of Trás-os-Montes and Alto Douro, Vila Real, Portugal; ^2^BioISI - Instituto de Biosistemas e Ciências Integrativas, Faculdade de Ciências, Universidade de Lisboa, Lisbon, Portugal; ^3^LEAF - Linking Landscape, Environment, Agriculture and Food-Research Center, Associated Laboratory TERRA, Instituto Superior de Agronomia, Universidade de Lisboa, Lisbon, Portugal; ^4^REQUIMTE, Instituto Superior de Engenharia do Porto, Porto, Portugal; ^5^Institute of Grapevine and Wine Sciences (ICVV), Spanish National Research Council (CSIC), University of La Rioja and Government of La Rioja, Logroño, Spain

**Keywords:** grapevine trunk diseases, detection, identification, molecular markers, PCR

## Abstract

Grapevine is regarded as a highly profitable culture, being well spread worldwide and mostly directed to the wine-producing industry. Practices to maintain the vineyard in healthy conditions are tenuous and are exacerbated due to abiotic and biotic stresses, where fungal grapevine trunk diseases (GTDs) play a major role. The abolishment of chemical treatments and the intensification of several management practices led to an uprise in GTD outbreaks. Symptomatology of GTDs is very similar among diseases, leading to underdevelopment of the vines and death in extreme scenarios. Disease progression is widely affected by biotic and abiotic factors, and the prevalence of the pathogens varies with country and region. In this review, the state-of-the-art regarding identification and detection of GTDs is vastly analyzed. Methods and protocols used for the identification of GTDs, which are currently rather limited, are highlighted. The main conclusion is the utter need for the development of new technologies to easily and precisely detect the presence of the pathogens related to GTDs, allowing to readily take phytosanitary measures and/or proceed to plant removal in order to establish better vineyard management practices. Moreover, new practices and methods of detection, identification, and quantification of infectious material would allow imposing greater control on nurseries and plant exportation, limiting the movement of infected vines and thus avoiding the propagation of fungal inoculum throughout wine regions.

## Grapevine biodiversity and cultural and economic value worldwide

**FIGURE 1:**
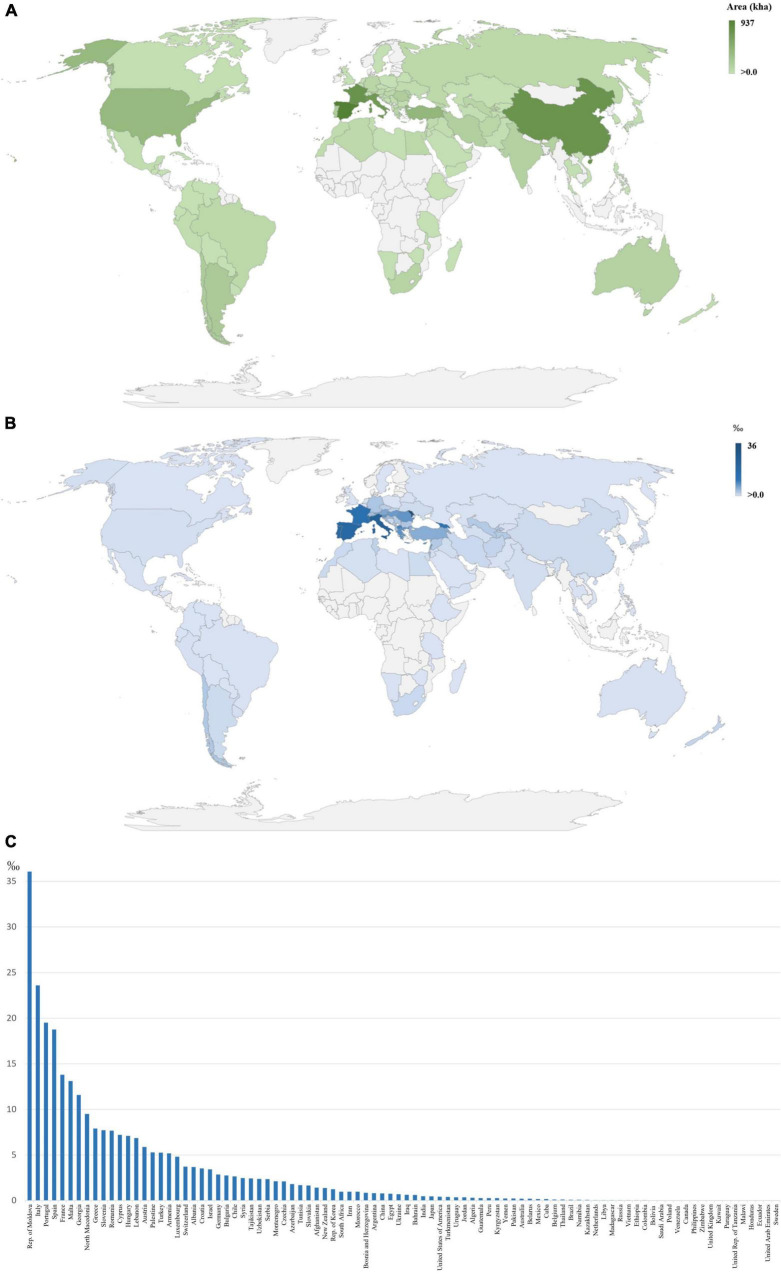
Geographical identification of vineyards (in kha) **(A)** and in permille (‰) **(B,C)**, comparing the dedicated grape production area to the total area of each country, with a visible cluster in European, Middle Eastern, and North African countries, mainly countries close to the Mediterranean Sea (FAOSTAT, 2019), where higher values are represented by darker tones.

Grapevine (*Vitis vinifera* L.) is distributed worldwide (Figure 1A) mainly due to its high adaptation capability. The genetic diversity found among grapevine varieties is very high, with around 10,000 estimated cultivars available in germplasm banks (This et al., 2006). This genetic diversity has been obtained through fast mutation events or by slow selection through sexual and asexual reproduction, with no particular emphasis on which has been the most prevalent (This et al., 2006). The selection of the most desirable traits has led to an economic and cultural impact on the wine-producing countries (This et al., 2006). In the interest to preserve this variability, wide germplasm collections have been established, containing several autochthonous cultivars of each country (This et al., 2006). The Mediterranean countries detain a big part of the biodiversity available within this species (Figures 1B,C), since currently, the grapevine has a huge cultural heritage apart from being economically very important for these wine-producing countries (This et al., 2006).

Wine production mobilizes high monetary values, with the global market reaching over 29 billion euros in 2020, mostly in the form of bottled wine (OIV, 2020). Grapevine is regarded as the most cultivated fruit crop worldwide, with nearly 7 million hectares planted (Figure 1A) and with yield values of more than 77 million tons (FAOSTAT, 2019). Most of the grape production is directed to the wine industry, where wine value increases with the implementation of traceability systems linked to regions with a denomination of origin systems (Pereira et al., 2018).

In this perspective, any factor that leads to yield decrease, quality loss, or vine disruption, combined with increased costs associated with vineyards maintenance, will greatly affect the economic comeback of producers. One of the main causes of productivity loss in grapevines is the occurrence of grape trunk diseases (GTDs), which can lead to vine death (Gramaje et al., 2018). The incidence of the disease has been increasing in the last few years due to new constraints imposed in vineyard management, e.g., the ban on sodium arsenate and cultural practices in vineyards with poor training and pruning systems, among others (Rubio and Garzón, 2011).

## Effects of grapevine trunk diseases

**FIGURE 2:**
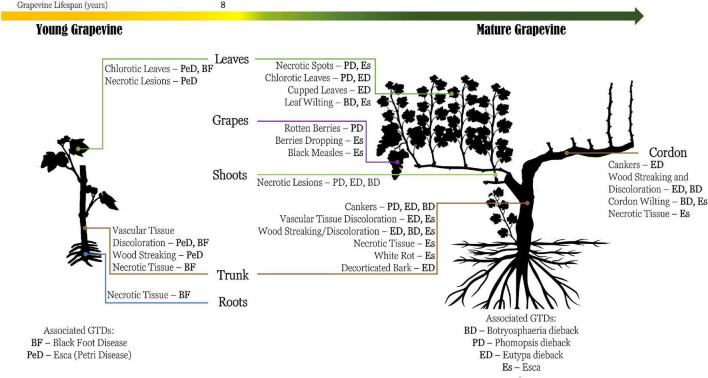
Schematic representation of the commonly known symptomology regarding grapevine trunk diseases (GTDs), based on the description of the reviewed literature. Generally, the main effect caused by GTDs is stunted development of the vines and necrosis, partially or completely, which can lead to the decay of the plant and consequently production loss. Esca (Es), Petri disease (belonging to Esca complex) (PeD), Botryosphaeria dieback (BD), Eutypa dieback (ED), Phomopsis dieback (PD), and black foot disease (BF).

Grapevine trunk diseases include an extensive group of symptomatic fungal infections that affect vineyards with damaging economic effects (Gramaje et al., 2018; Mundy et al., 2018). Several distinctive diseases have already been vastly reported with detailed symptomatology description (Figure 2). However, several conditions, such as climate and grape vineyard management, may lead to differences in the symptomology patterns of GTDs. According to the age of grapevines, some GTDs are more likely to be detrimental to others (e.g., black foot disease and Petri Disease affect mainly young vines, in contrast to Eutypa dieback, Phomopsis dieback, and esca that affect mature vines). Additionally, some specific characteristics associated with the grapevine genotype may influence the susceptibility of the grapevine cultivar to certain GTDs (Mondello et al., 2018). Furthermore, grapevines can be affected simultaneously by several GTDs, thus hampering their identification and vineyard management (Agustí-Brisach and Armengol, 2013; Gramaje et al., 2018).

Several mechanisms have been used by the fungal agents of GTDs for disease dispersion; among them, dispersion by fungal spores is easily achieved among vines as a result of rain splash, wind, insects, or due to the presence of pathogenic fungal inoculum in the soil (Cloete et al., 2011; Trouillas et al., 2011; Gramaje et al., 2018; Moyo et al., 2019; Nerva et al., 2019). Vine colonization by soil microorganisms is a recurring effect that can affect the expression of grapevine trunk disease; therefore, soil microbiome analysis also provides several clues for GTD expression, mostly for pathogens belonging to Esca and black foot disease (Giménez-Jaime et al., 2006; Nerva et al., 2019). However, each GTD pathogen has a preferential method for overall transmission. Furthermore, the debris left on the vineyard, mechanical and human actions, and even host species in the proximity of vineyards increase the risk of outbreaks (Cloete et al., 2011; Trouillas et al., 2011; Elena and Luque, 2016; Moyo et al., 2019). These transmission and infection mechanisms are highly dependent on external conditions, such as the season of the year and the edapho-climatic conditions (Songy et al., 2019; Hrycan et al., 2020).

Such a destructive complex of diseases has a considerable impact on fruit yield and quality and hence on the economic value of the product, leading to great economic losses in wine-producing regions and countries (Gramaje et al., 2018). Lately, due to new practices and abolishment of some chemical treatments, the number of outbreaks has increased, as has GTD-associated damage in the vineyards, which seem to be transversal to all the GTDs further mentioned.

## Grapevine trunk disease (GTD)-associated pathogens and related symptomology

### Esca complex and Petri disease

Esca is a complex disease that englobes several syndromes previously identified as GTDs, such as esca (as a disease), grapevine leaf and stripe disease, apoplexy, black measles, white rot, and Petri disease (Bertsch et al., 2013; Choueiri et al., 2014; Cloete et al., 2015; Gramaje et al., 2018; Claverie et al., 2020). The causal agents of the esca disease are mainly the same within syndromes, with some exceptions further enlightened. To date, fungal species belonging to genera *Phaeoacremonium* and *Phaeomoniella* have been recognized as causal agents of every disease within the esca complex through classic and molecular marker approaches. Several species of *Phaeoacremonium* that are pathogenic to grapevine have been reported so far (Supplementary Information 1). Fungal pathogens *Phaeoacremonium minimum* and *Phaeomoniella chlamydospora* are considered to be the most prevalent esca pathogens in the wine-producing countries, where esca has been reported in several vineyards worldwide. However, several other fungal pathogens were found to have a negative impact on grapevines with symptoms associated with esca. However, they are considered as secondary pathogens, as they normally produce fewer symptoms. Additionally, microbial interactions between pathogenic fungal species and these secondary pathogenic species may lead to symptom aggravation or imbalance reducing plant vigor (del Frari et al., 2021; Haidar et al., 2021). Some of the secondary pathogens are related to every Esca-related syndrome with the exception of Petri disease, and therefore are found only in mature vines (Supplementary Information 1). On the other hand, there are putative secondary pathogens that are, at this point, uniquely related to Petri disease (Supplementary Information 1), thus affecting only young vines (Gramaje et al., 2018; Raimondo et al., 2019; Mondello et al., 2020). However, it can potentially lead to slow decay of the vine over the years, and thus might also affect mature vines simultaneously as other Esca pathogens (Travadon et al., 2015).

The ultimate outcome of this disease, whether occurring rapidly or not (Almendros et al., 2019), is the decline of apoplexy of grapevine, either partially or completely, leading to the removal of the plant as a sanitary measure (Mondello et al., 2018). These symptoms appear mostly during the summer months, leading to a seemingly unavoidable decay of the vine. The symptoms might be perceptible for years and slow acting, and manifest as the chronic form of the esca disease or grapevine leaf and stripe disease, which starts normally at an earlier developmental stage of the vine. However, the acute and apoplectic form of esca is a fast attacking form, leading to a sudden vine decay, and is largely influenced by the meteorological conditions during the spring and early summer months (van Niekerk et al., 2011; Arzanlou et al., 2013a; Guérin-Dubrana et al., 2013, 2019). Berry and foliar symptoms can be caused by phytotoxins, which are produced by fungal pathogens or due to the blockage of systemic vessels (Brown et al., 2020). The notion that “esca proper” only infects mature vines is widely accepted; however, young grapevines have also been reported to be targeted by esca pathogens (Edwards and Pascoe, 2004).

Petri disease affects young vines, usually under 5–8 years old (Gramaje and Armengol, 2011; Cloete et al., 2015; Baranek et al., 2018; Hrycan et al., 2020). Its symptoms are close to black foot symptoms, and they lead the vine to a stunted growth with short aerial organs and delayed budding, which, in severe cases, ends up in apoplectic events of the plant (Edwards and Pascoe, 2004; Sosnowski et al., 2007a; Arzanlou et al., 2013a; Almendros et al., 2019). Also, symptoms observed in young vines affected by Petri disease might be due to malnourishment of the aerial parts due to the blockage of the systemic vessels (Castillo-Pando et al., 2001; Arzanlou et al., 2013a) and/or due to poor development of the host, which further influences the parenchyma density and host anatomy leading to a diminished innate immunity (Claverie et al., 2020). Moreover, the accumulation of toxins and secondary metabolites produced by the pathogens has a fundamental role in pathogenicity (Andolfi et al., 2011; Arzanlou et al., 2013a). The etiology of Petri disease is considered to be shared with the esca complex and its syndromes, whereas several wood pathogenic fungal species are correlated with the appearance of both these GTDs (Edwards and Pascoe, 2004; da Silva et al., 2017; González-Domínguez et al., 2020).

## Black foot disease

Black foot disease commonly affects grapevines at an early age and infects new cuttings or grafts (Agustí-Brisach and Armengol, 2013). The symptoms of this pathology are well-established and used for its identification. It is mostly characterized by the infection of the roots and rootstocks (Gramaje and Armengol, 2011), affecting the vascular system development of young grapevines and leading to reduced plant vigor and sometimes plant death as a result of reduced structures, such as internodes and leaves, and chlorosis. Also, the affected wood tissue becomes dark brown to black colored, which further gives the plant a dark and necrotic appearance immediately above the soil (Halleen et al., 2006; Gramaje et al., 2018). It disturbs, devastatingly, new grapevines under 8 years old, leading rapidly to their death, and black foot also ravages through older plants, although at a slower rate (Halleen et al., 2006), causing economic damages when vineyards are affected by the causal pathogens of black foot disease (Pathrose et al., 2014; Mondello et al., 2018).

According to the current knowledge, the causal agents of this disease belong to the genera *Campylocarpon*, *Cylindrocladiella*, *Ilyonectria*, *Dactylonectria*, *Neonectria, Thelonectria, and Pleiocarpon* (Supplementary Information 1) and its anamorphic appearances (Halleen et al., 2006), most of them being previously reported as belonging to the genus “*Cylindrocarpon*” with further classification into *Ilyonectria*, *Neonectria*, and *Dactylonectria* genera, as shown in Mycobank (2021) (www.mycobank.org).

The most pathogenic status has been associated with the species *Dactylonectria macrodydima* (Alaniz et al., 2009) and *Dactylonectria novozelandica* (Berlanas et al., 2020), which are found in the Spanish vineyards. However, such classifications may be dependent on environmental conditions that are believed to affect fungal development and the lack of analyses which have limited the search to a few species, thought to be the most prevalent and the easiest to be isolated in the studied areas.

So far, there are some reports which demonstrate several fungi as causal agents of black foot in grapevine. All these fungi are considered to be saprophytes, maintaining a lifecycle where infectious particles can be attached to grapevine debris and/or be present in the soil (Agustí-Brisach and Armengol, 2013; Carlucci et al., 2017) and thus affecting newly planted specimens. Also, nurseries can be focal points for black foot infection (Halleen et al., 2006).

Despite not being deeply studied, reports show the worldwide distribution of this genus (Agustí-Brisach and Armengol, 2013) with the distinct prevalence of some of these pathogens in distinct locations: *Dactylonectria macrodydima* in South Africa (Langenhoven et al., 2018), and *Dactylonectria torresensis* in Italy (Carlucci et al., 2017), Portugal (Reis et al., 2013), and Spain (Berlanas et al., 2017, 2020), which seems to be the most prevalent species.

## Eutypa dieback

The appearance of cankers in grapevines is a symptom that shows that the vine might be affected by Eutypa dieback; nevertheless, it occurs in the late stage of this disease (Gramaje et al., 2018). Previous symptoms include the appearance of necrotic tissue in shoots, spurs, and at the margin of wedge-shaped cankers affecting vine trunks and cordons; wood streaking and discoloration; and decorticated bark and wood (Trouillas and Gubler, 2010b). Overall, Eutypa dieback-related pathogens colonize the vascular system of the vine (Sosnowski et al., 2008), reducing the vegetative growth and eventually leading to vine death (Baranek et al., 2018). Infection occurs in vines that have open and fresh wounds, mostly related to pruning types or vine management with symptoms appearing in older vines (Sosnowski et al., 2008; Gramaje et al., 2018). Spore dispersal occurs through wind and favorable conditions of high humidity and low temperature (Sosnowski et al., 2007b), with more prevalence in autumn and winter. However, rain is not mandatory for spore release (Úrbez-Torres et al., 2020). Inoculum sources of Eutypa dieback pathogenic fungi can be among other economically interesting plant species, such as apricot, maple, willow, and wild species (*Tilia* and *Lonicera*), where *Eutypa lata* is the most reported. Thus, cases of cross-contamination are observed, without any decrease in pathogenicity and no host specificity (Travadon et al., 2012; Travadon and Baumgartner, 2015; Moyo et al., 2019).

The first struggle for identification and control of this disease is due to the late appearance of the symptoms, which are delayed for 1–8 years after the infection, hence affecting mature vines (Sosnowski et al., 2007a), and additionally, they are influenced by the climatic conditions (Sosnowski et al., 2007b). Identification of other pathogens and their classification has been tenuous and hard work, since several distinct genera are involved in Eutypa dieback in grapevine (Trouillas and Gubler, 2010a; Mehrabi et al., 2016). *Eutypa lata* is considered as the most prevalent pathogenic fungus that causes Eutypa dieback; however, other Diatrypaceae species have a greater role than previously thought (Úrbez-Torres et al., 2020). Also, *Eutypa lata* is considered as the only species within all Eutypa dieback pathogenic fungi that cause foliar symptoms (Sosnowski et al., 2007a). It is responsible for the production of phytotoxins and secondary metabolites that are harmful to grapevine growth (Tey-Rulh et al., 1991; Molyneux et al., 2002; Mahoney et al., 2003) and is indicated as the main reason for the appearance of foliar symptoms (Sosnowski et al., 2007a).

Even though the most common and primarily recognized as causal agent worldwide is *Eutypa lata*, there are several other fungal species that are less prevalent in Eutypa dieback disease, and they belong to the genera *Eutypa*, *Eutypella, Diatrype, Diatrypella, Cryptosphaeria, Cryptovalsa, Anthostoma*, and *Peroneutypa* (Supplementary Information 1). Although some studies indicate that some of these other pathogenic species have similar virulence (Trouillas and Gubler, 2010b), so far, several fungal species that are associated with Eutypa dieback in grapevine have been discovered in grapevine tissues (Supplementary Information 1). However, some are not associated with symptoms in grapevine, despite being isolated from grapevine wounds and cankers, such as *Diatrype oregonensis* and *Diatrype whitmanensis* (Trouillas and Gubler, 2010b). Additionally, other unidentified species within the *Eutypa*, *Diatrype*, *Diatrypella, Cryptosphaeria*, and *Peroneutypa* genera were also identified as pathogenic for grapevine (Luque et al., 2012; Pitt et al., 2013; Rolshausen et al., 2014; Paolinelli-Alfonso et al., 2015; Moyo et al., 2019). Nevertheless, even when molecular markers were used for fungal discrimination, a reliable identification was not possible. At best, it is only possible to assert a relation of proximity to other species, such as *Eutypa* sp. that was somewhat closer to *Eutypa tetragona* (Luque et al., 2012); *Eutypella* sp. group 1 is closely related to *Eutypella scoparia*; *Eutypella* sp. group 2 is closely related to *Eutypella vitis;* and *Eutypella* sp. group 4 is closely related to *Eutypella leprosa* and several *Diatrypella* sp. which conjugate closely as a putative single species complex which englobes *Diatrypella verrucaeformis* (Trouillas et al., 2010). Further studies are needed to fully unravel the fungal pathogens of Eutypa dieback and its relatedness, since some species might not be well identified, as it happens with some isolates of *Cryptovalsa rabenhorstii* that seem to be closely related to *Eutypella* species (Paolinelli-Alfonso et al., 2015).

## Botryosphaeria dieback

Botryosphaeria dieback is another GTD that is related to mature vines, e.g., over 8 years old (van Niekerk et al., 2004; Gramaje et al., 2018), which also infects young vines and nurseries (Giménez-Jaime et al., 2006; Ammad et al., 2014; Billones-Baaijens et al., 2015), showing few (bud mortality and failed graft junctions) to no symptoms (van Niekerk et al., 2006; Billones-Baaijens et al., 2015). Classified as a disease of interest in the early 21st century (Billones-Baaijens and Savocchia, 2018), Botryosphaeria dieback was initially mistaken as Eutypa dieback and Phomopsis dieback due to the similarity of symptoms, such as cankers, wood staining, and wilting of plant structures in mature vines (Phillips, 1998; van Niekerk et al., 2004; Billones-Baaijens and Savocchia, 2018; Gramaje et al., 2018). Misclassification of Botryosphaeria dieback, as other known GTDs, was also due to several difficulties related to the fungal identification, since morphologic markers are limited and shared between several fungal species, and sexual morph stages are seldom discovered in natural conditions and hardly obtained in culture (van Niekerk et al., 2004). Molecular technologies helped to unravel this fungal family, as explained further.

Currently, considered as one of the fungal diseases in the GTD complex, several fungal species associated with Botryosphaeria dieback are considered to be pathogens for grapevines, all belonging to Botryosphaeriaceae family (Billones-Baaijens and Savocchia, 2018), including *Botryosphaeria*, *Diplodia*, *Lasiodiplodia*, *Neofusicoccum*, *Neoscytalidium*, *Phaeobotryosphaeria*, and *Dothiorella* genera (Supplementary Information 1), the most prevalent being *Botryosphaeria dothidea*, *Diplodia seriata*, *Lasiodiplodia theobromae*, and *Neofusicoccum parvum* (Kovács et al., 2017; Billones-Baaijens and Savocchia, 2018). The last two species are highly pathogenic toward grapevine with some variance due to edapho-climatic conditions (Úrbez-Torres and Gubler, 2009). So far, *Neofusicoccum* genus is considered to be more pathogenic when compared to *Diplodia* species (Amponsah et al., 2011), although some Botryosphaeriaceae fungal species may affect grapevine more extensively than others (Ramírez et al., 2018).

Potential inoculum sources are prevenient of other plant species that are in the proximity of the vineyard, for instance, blueberry, broom, willow, cherry, oak, plum, apple, pine, olive, and lemon wood (Amponsah et al., 2011). However, nursery infections of vine cuttings and grafting procedures of the plants also occur extensively, adding to another level of difficulty to overcome Botryosphaeria dieback (Billones-Baaijens et al., 2013b).

Additionally, severe environmental conditions affect fungal development and disease progression. For example, when grapevines are subjected to drought conditions, there is an enhancement of Botryosphaeria dieback symptoms caused by *Neofusicoccum parvum*, as suggested by Galarneau et al. (2019). Also, the pathogenic status of Botryosphaeria dieback pathogens may be related to the production of phytotoxins and secondary metabolites, as demonstrated in the isolates of *Diplodia seriata, Neofusicoccum parvum, Neofusicoccum luteum, Dothioriella viticola*, and *Botryosphaeria dothidea* (Martos et al., 2008).

## Phomopsis dieback

The genus *Diaporthe* [formerly known by its asexual morph *Phomopsis* (Úrbez-Torres et al., 2013)] is responsible for a well-known GTD, commonly identified as Phomopsis dieback (Gramaje et al., 2018). Initial etiology can be prone to misidentifications, due to several ambiguous classifications of the causal pathogens, mainly due to morphological characteristics, such as plasticity, host association, and asexual morph/sexual morph distinction (Udayanga et al., 2012; Úrbez-Torres et al., 2013). The main causal agent of Phomopsis dieback is *Diaporthe ampelina* (formerly *Phomopsis viticola*) (Dissanayake et al., 2015; Lawrence et al., 2015). Now, due to the advent of molecular technologies, several causal agents of Phomopsis dieback have been described and reported (Supplementary Information 1). However, *Diaporthe perjuncta* has been reported to be non-pathogenic for grapevines in Australia (Rawnsley et al., 2004), in contrast with pathogenic isolates obtained in Portuguese vineyards (Phillips, 1998). Still, phylogeny studies have reported closely related species, which are grouped in species complexes accordingly, i.e., *D. eres* complex which englobes several *Diaporthe* spp. being, so far, only *D. eres* species associated with a grapevine (Yang et al., 2018). Also, other *Diaporthe* spp. can be endophytic and isolated from grapevine without apparent symptomology associated, such as *Diaporthe bohemiae* (Guarnaccia et al., 2018). Despite not being found, *D. bohemiae* can have an opportunistic behavior toward grapevine, like other *Diaporthe* spp. have toward herbaceous weeds and fruit trees, e.g., *D. foeniculina* (Lesuthu et al., 2019; Manawasinghe et al., 2019).

This disease affects mature grapevine wood and presents clear symptoms which are related to the appearance of perennial cankers in the vine, which is one of its main characteristic symptoms, as described by several authors (Nita et al., 2006a; Gramaje et al., 2018; Úrbez-Torres et al., 2013; Manawasinghe et al., 2019). Additionally, it leads to a budding reduction and withering when infected (Úrbez-Torres et al., 2013). Infection is simplified with the existence of open and fresh wounds, mostly related to pruning practices (Travadon et al., 2013). Nita et al. (2008) showed that Phomopsis dieback has distinct incidences between plant structures with lower variation caused by environmental conditions and location.

Phomopsis cane and leaf spot (also Excoriosis) is a classification for a syndrome caused by the same fungal agents that are responsible for Phomopsis dieback, being the symptoms distinctive between these two syndromes, where *D. ampelina* has a particular incidence (Baumgartner et al., 2013). Some authors englobe both syndromes under the same disease (Manawasinghe et al., 2019). Plants with preceding Phomopsis cane and leaf spot symptoms are more susceptible to Phomopsis dieback (Baumgartner et al., 2013).

Phomopsis dieback is considered as a monocyclic disease, since mostly the primary inoculum causes the infection, and symptoms appear during the growing season under favorable environmental conditions (Anco et al., 2012). Inoculum dispersal is produced by rain (Nita et al., 2006a,b), dispersing conidia from infected plant organs (Travadon et al., 2013) and debris (Anco et al., 2012) to healthy ones, possibly with a limited and short range of dispersal (Nita et al., 2006a).

Similar to other GTDs, Phomopsis dieback has a worldwide distribution with a high negative economic effect, and is caused by several fungi. To attest this occurrence, reports show that *Diaporthe eres* is more prevalent in Europe and Israel (Guarnaccia et al., 2018), as well as in China (Manawasinghe et al., 2019). In contrast, in Spain, the occurrence rates of *D. ampelina* and *D. baccae* are similar to that of *D. eres* (Guarnaccia et al., 2018). These differences may be explained by climacteric and environmental conditions. However, presently, there are no reports that confirm any explanation regarding this subject.

## Cytospora canker

*Cytospora* genus is further regarded as a causal agent of another canker-causing GTD; however, in this review, we only mention it as a GTD-causing pathogen, since there are few studies reporting it (Tomoiaga et al., 2009). Despite being under-studied in grapevine, some species belonging to *Cytospora* genus have already been identified, being of major significance in grapevine, since they might be pathogenic, thus reducing the yield and viability of the vines (Supplementary Information 1) (Lawrence et al., 2017). The most characteristic symptoms of this disease are the appearance of cankers in vines and its decline (Lawrence et al., 2017).

## Fungal interactions with grapevine trunk disease (GTD) pathogens

The identification of fungal pathogens is not fully representative of the disease. Recent studies performed on symptomatic grapevines samples, where pathogens have been identified, revealed the presence of other fungal species, not considered common saprophytes, that affected disease progression (Supplementary Information 1), such as *Schizophyllum commune* in affected vines co-inoculated with *Lasiodiplodia theobromae* and *Neofusicoccum parvum* (Rezgui et al., 2018); *Neopestalotiopsis, Pestalotiopsis*, and *Truncatella* genus in vines affected either by Botryosphaeria dieback, Phomopsis dieback, or Esca (Maharachchikumbura et al., 2016); and *Roesleria*, and *Sphaeropsis* in GTD-affected vines (Tomoiaga et al., 2009). These fungal species create a complex pathogenic microbiome that can be relevant to the deeper understanding of the GTD complex, even as the causal or secondary infection agents of GTDs, as seems to occur with *Seimatosporium vitis* in symptomatic vines affected by *Phaeoacremonium* spp. and *Fomitiporia mediterranea* (Amarloo et al., 2020); *Schizophyllum commune* in vines infected with *Lasiodiplodia theobromae* and *Neofusicoccum parvum* (Rezgui et al., 2018); and other *Seimatosporium* spp. (*Seimatosporium vitifusiforme* and *Seimatosporium luteosporum*) in co-inoculations with other GTD pathogens in vines (Lawrence et al., 2018). However, most of these interactions between fungi and grapevine are not well-documented, as in the case of *Sporocadus, Pestalotiopsis, Dendrothyrium*, and *Truncatella*, among other genera, whose role in the GTD process and/or infection is still not clarified (Arzanlou et al., 2013b; Abed-Ashtiani et al., 2019; Mundy et al., 2020). Additionally, studies performed in Hungary showed that fungi genera are emerging continuously, affecting canonically other woody hosts, such as apple and pear, that may cause symptoms similar to those observed in GTDs, namely, *Pseudofabraea, Phlyctema, Parafabraea*, and *Neofabraea*, thus increasing the intricacy of this disease complex (Lengyel et al., 2020). Furthermore, fungal identification from wounded tissues may show activity from saprobic or endophytic fungi upon tissues with less integrity that might have no role in the infectious and colonization processes (*e.g., Trametes versicolor* in necrotic tissue of GTD-affected vines) (Mundy et al., 2020).

## Methods for grapevine trunk disease (GTD) assessment

### Classical methods

Grapevine trunk diseases are detectable when grapevines show visible symptoms, and GTD identification is based on symptom detection in live plants with further morphological identification of the isolates in culture (Andolfi et al., 2011). However, they lead to misconceptions and misclassifications due to morphological plasticity and overlapping (Acero et al., 2004; Mehrabi et al., 2016).

Therefore, due to the nature of such pathologies, the setting of symptoms is a signal of an intermediate to the advanced stage of the disease, and economic losses are therefore already unavoidable (Almeida et al., 2020).

Methods that rely on fungal culture have several drawbacks, which include the time and costs associated, the need for experienced technicians, the destructive sample collection methods, and the incapability to perform wide and high-throughput analysis. Also, fungal identification is not easily accomplished just by culture, since several characteristics overlap within fungal species and genera (Gramaje and Armengol, 2011; Eichmeier et al., 2018; Morales-Cruz et al., 2018b). So far, methods suitable for early detection are scarcely used to the detriment of classical identification, posing a delay in the early mitigation practices required to control and contain GTDs. Hence, resourcing other methods, such as chemical, serological, and DNA-based methodologies, would help to avoid classification errors and further allow a more rapid and accurate diagnosis of the pathogens affecting grapevines. Nonetheless, some technological limitations are persistent in some situations (Lecomte et al., 2000; Rolshausen et al., 2004; Lardner et al., 2005).

Nevertheless, microorganism culture also brings new tools to understand microbial interactions as well as new ways to control pathogens, thus leading to a lighter or inexistent GTD symptomology by having an antagonistic role against GTD causal agents (Niem et al., 2020).

### Chemical approaches

The manifestation of the symptoms may be due to the secondary metabolites or toxins produced by the pathogens. Phytotoxin production by GTD-related pathogens has already been reported in infected vines, which are as follows:

- *Diaporthe eres* produces 4-hydroxybenzaldehyde, 4-hydroxybenzoic acid, nectriapyrone, p-cresol, and tyrosol, which induce Phomopsis dieback symptoms in grapevine (Reveglia et al., 2019);
- *Neofusicoccum parvum*, in plate culture, produced several metabolites and most of them (-)-terremutin, (+)-*epi*-sphaeropsidon, (+)-(6R,7S)-dia-asperlin, (-)-(3R,4S)-*trans*-4-hidroxymellein, (-)-(3R,4S)-*cis*-4-hidroxymellein, (-)-(R)-3-hidroxymellein, and (-)-mellein) seem to have phytotoxic activities against grapevine in leaf disk assay (Abou-Mansour et al., 2015), whereas (-)-mellein is also produced by other Botryosphaeriaceae fungal species, namely, *Diplodia mutila, Diplodia seriata, Neofusicoccum australe*, and *Neofusicoccum luteum* (Masi et al., 2020b);
- *Neofusicoccum luteum* produces luteopyroxin, neoanthraquinone, luteoxepinone, and tyrosol with potential phytotoxic activities (Masi et al., 2020a);
- isolates of *Phaeomoniella* and *Phaeoacremonium* genera produce several metabolites that have a nefarious impact on grapevine growth and development, such as isosclerone and scytalone (Andolfi et al., 2011; Bertsch et al., 2013);
- *Fomitiporia mediterranea* produces metabolites with nefarious effect on grapevine structures, and, at least, 4-hydroxybenzaldehyde is shared between the genera *Phaeoacremonium* and *Phaeomoniella* (Andolfi et al., 2011).

Even though several metabolites have been identified, this may not be sufficiently reliable to stand alone as a pathogen identification method, since the metabolic profile obtained consists of interactions between plant/pathogens and endophytic microorganisms (Amponsah et al., 2011; Azevedo-Nogueira et al., 2020), and thus may not be exclusive of the pathogen itself. Also, variation in the secreted metabolites and proteins can also be related to differential pathogenicity, as verified in the proteomic analysis of *Neofusicoccum parvum* and *Diplodia seriata* isolates, whose proteomic profiles are distinct and seem to influence the aggressiveness of these pathogens against *Vitis vinifera* cv. Chardonnay *calli*, with *Diplodia seriata* being less aggressive (Benard-Gellon et al., 2015). In addition, the metabolites produced can also be affected by the edapho-climatic conditions (Azevedo-Nogueira et al., 2020; Hrycan et al., 2020). Furthermore, secondary toxic metabolites are shared between several fungal pathogens of distinct GTDs, such as tyrosol, isosclerone, and 4-hydroxybenzaldehyde (Andolfi et al., 2011; Masi et al., 2018). Hence, molecular diagnostic methods, using DNA amplification (polymerase chain reaction (PCR)-based), are the most indicated to attain such objectives, since they are able to overcome the several difficulties found when using more standard techniques, such as morphological- and chemical-based ones (Azevedo-Nogueira et al., 2020).

### Endpoint polymerase chain reaction (PCR) and PCR-based techniques

So far, the identification of several fungi related to GTDs is made by amplification and sequencing of genomic fragments, mainly *ITS* and *rDNA* (large ribosomal subunit and small ribosomal subunit) (Lecomte et al., 2000; Armengol et al., 2001; Acero et al., 2004; Taylor et al., 2005; van Niekerk et al., 2005; Cunnington et al., 2007; Martin and Cobos, 2007; Abreo et al., 2010; O’Gorman et al., 2010; Pitt et al., 2010; Amponsah et al., 2011; Qiu et al., 2011; Wunderlich et al., 2011; Arzanlou et al., 2013b; Ammad et al., 2014; Mehrabi et al., 2016; Chebil et al., 2017; Kovács et al., 2017; Rezgui et al., 2018; Narmani and Arzanlou, 2019; Mondello et al., 2020; Salazar et al., 2020) and others. In order to obtain a greater definition, other markers, such as *Tub2*, *His3*, *Act*, and *Tef1* (van Niekerk et al., 2004; Schilder et al., 2005; Mohammadi et al., 2009; Besoain et al., 2013; Berlanas et al., 2017; da Silva et al., 2017; Lawrence et al., 2017; Baranek et al., 2018; Amarloo et al., 2020), have been used. Some fungal families (Hymenochaetaceae and Phaeomoniellaceae) related to Esca disease can be correctly identified by just using the ITS fragment sequencing analysis (Fischer and Kassemeyer, 2003). However, to ensure a more reliable identification, the simultaneous analysis of several genomic regions is made, bringing a higher resolution capacity to problematic and difficult identifications, as in the cases of black foot disease (Cabral et al., 2012), Eutypa dieback (Rolshausen et al., 2014), Botryosphaeria dieback (Yan et al., 2013), and Phomopsis dieback (Guarnaccia et al., 2018). To a lesser extent, other molecular methods are also used to detect and identify fungal pathogens, such as nested PCR for black foot disease in soils, plants, and grafted cuttings (Nascimento et al., 2001; Agustí-Brisach et al., 2013, 2014), Botryosphaeria dieback pathogens *in planta* (Spagnolo et al., 2011), and Eutypa dieback pathogens from infected vines (Catal et al., 2007); microsatellite analysis for identification of fungal isolates of *Eutypa lata* (Baumgartner et al., 2009); inter-simple sequence repeats and/or random amplified polymorphic DNA amplification for black foot pathogens from pure fungal isolates (Alaniz et al., 2009; Reis et al., 2013) and *Kalmusia variispora* (Abed-Ashtiani et al., 2019); restriction fragment length polymorphism for the identification of Botryosphaeriaceae fungi (Billones-Baaijens et al., 2013a), *Eutypa lata* (Rolshausen et al., 2004), and several fungi (Martin and Cobos, 2007) from pure cultured isolates; and single-strand conformation polymorphism for Botryosphaeria fungal identification (Billones-Baaijens et al., 2013b).

**FIGURE 3:**
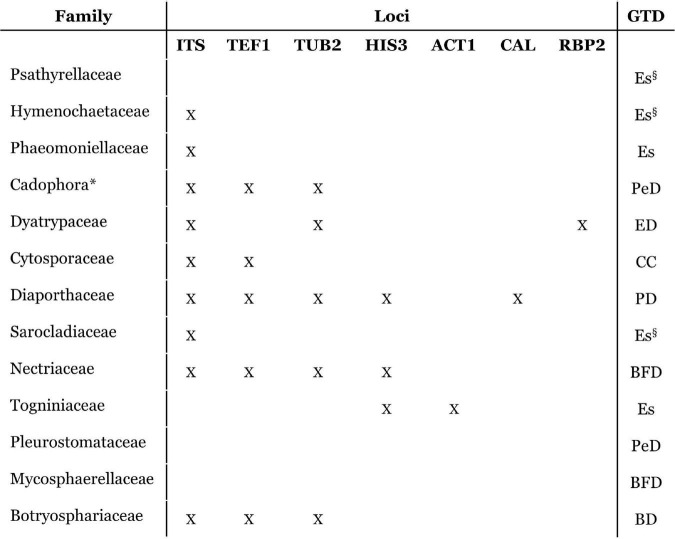
Schematic representation of the accepted grapevine trunk disease (GTD)-causing fungal families, indicating the *loci* that are usually used for species identification within each fungal family (Cabral et al., 2012; Arzanlou et al., 2013a; Yan et al., 2013; Rolshausen et al., 2014; Travadon et al., 2015; Lawrence et al., 2017; Dissanayake et al., 2018; Guarnaccia et al., 2018; Gramaje et al., 2019) in those in which these are applicable. Several families need multi-*loci* analysis to accurately identify species. Esca (Es), Petri disease (belonging to Esca complex) (PeD), Botryosphaeria dieback (BD), Eutypa dieback (ED), Phomopsis dieback (PD), black foot disease (BF), and Cytospora Canker (CC). *Cadophora* is a fungal genus and is therefore indicated (*) for easier comparison to other fungal families. Families/genera which are not recognized to be involved in Petri disease, and therefore do not affect young vines, but have a role in esca-affected mature vines are indicated (Es^§^).

Nowadays, sequencing a panel of several genomic regions is required to achieve a clear taxonomic identification at the species level for several GTDs (Figure 3). These results are obtained by means of pure culture isolates of the infecting fungi, which are more reliable and accurate than the analysis of purely morphological characteristics. Nevertheless, culture isolation is biased, as microorganisms that have slower growth rates, smaller inoculum, less competitive behavior, and biotrophic lifestyles are harder to isolate, leading to an identification bias and leaving several fungal taxa left unidentified (Dissanayake et al., 2018).

### Quantitative polymerase chain reaction (PCR) and droplet digital PCR

Methods that are more robust with a wider and faster sample analysis are also used to obtain reliable results, as demonstrated in *Eutypa lata* and *Diplodia* spp. detection by quantitative PCR (qPCR) in pruning wounds (Pouzoulet et al., 2017); in *Eutypa lata* detection and quantification in grapevine wood samples (Moisy et al., 2017); black foot disease pathogen in nursery soil samples by qPCR (Agustí-Brisach et al., 2014; Langenhoven et al., 2018); *Cadophora luteo-olivacea* from vineyard soils and nursery vine stocks by qPCR and TaqMan^®^-based assay with droplet digital PCR (ddPCR) (Maldonado-González et al., 2020); *Phaeomoniella chlamydospora*, an Esca-related pathogen, in several steps of the nursery propagation process, by a TaqMan^®^ qPCR assay (Edwards et al., 2007); and quantification and monitoring of the field samples throughout the year by qPCR (González-Domínguez et al., 2020); other Esca-related pathogen (*Phaeomoniella chlamydospora* and *Phaeoacremonium aleophilum*) quantitation by qPCR in wood samples (Pouzoulet et al., 2013); Botryosphaeriaceae family fungi identification and quantification by hydrolysis qPCR (Billones-Baaijens et al., 2018); and *Ilyonectria liriodendri* by qPCR and ddPCR (del Pilar Martínez-Diz et al., 2020). Also, the identification and quantification of the microbiomes of roots are conceivable, making it possible to associate soil characteristics and vine rootstocks with the prevalence of some microbial agents (Berlanas et al., 2019).

**FIGURE 4:**
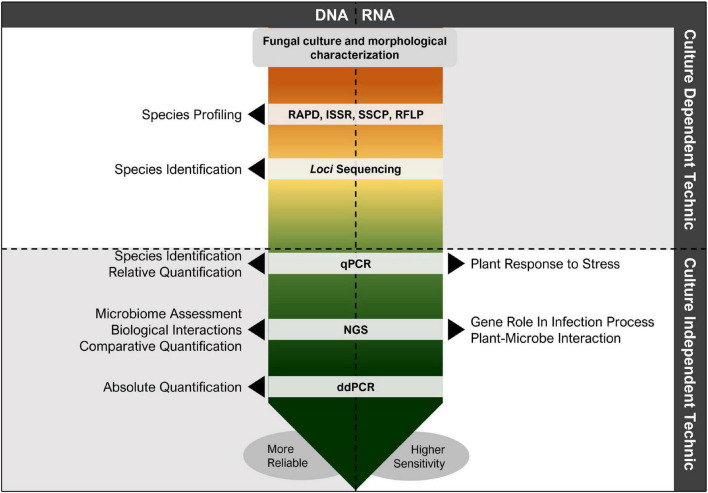
Diagram indicating the most used technics for grapevine trunk disease (GTD) assessment. These technics can have several outcomes, however, can be used simultaneously to overcome the limitations of the individual techniques. Nonetheless, more protocols have been developed aiming at more reliable and sensitive GTD assessment and studies, where species can be rapidly detected and interactions between fungal pathogens, grapevine and grapevine varieties, microbiome (fungi, bacteria, and virus), and environmental conditions can be easily assessed.

These methodologies are limited to the knowledge obtained from *loci* sequencing, since several GTD-related fungal species are identified by several *loci*, such as with endpoint PCR. Nonetheless, qPCR and ddPCR are incrementally more sensitive, allowing to detect lower quantity of inoculum (Pouzoulet et al., 2013, 2017; Agustí-Brisach et al., 2014; Maldonado-González et al., 2020), proving to be an excellent and accurate tool to be used, and retrieving highly satisfactory results when compared to other technologies (Figure 4), e.g., next-generation sequencing (Morales-Cruz et al., 2018b; Saccà et al., 2019).

## Next-generation sequencing

Modern technologies, based on massive next-generation sequencing (NGS) analysis (Gramaje et al., 2018), lead to faster and more reliable identifications, surpassing struggles that first existed and increasing our knowledge by bypassing the culture step and leading to the advent of culture-independent techniques. The main benefit of such methods is the wide microbiome analysis in each sample, allowing it to be applied in several samples simultaneously without the need to isolate the detected agents. These methodologies also allow to identify and relatively quantify the presence of each microbial agent (Eichmeier et al., 2018; Morales-Cruz et al., 2018b; Nerva et al., 2022). Additionally, distinct NGS approaches can have several outcomes, such as the perception of the role of the genes in the infectious process by RNA sequencing (Morales-Cruz et al., 2015, 2018a; Paolinelli et al., 2022), the prevalence of certain pathogens in distinct environments and grapevine cultivars by environmental microbiome metasequencing (Maree et al., 2012; Eichmeier et al., 2018; del Frari et al., 2019; Azevedo-Silva et al., 2021), and the role of other microbial agents toward GTD-causing fungal pathogens (Berlanas et al., 2019; Saccà et al., 2019; Niem et al., 2020; Bekris et al., 2021; Cobos et al., 2022).

It is also of great relevance that pathogen detection and identification can be performed without destroying and endangering the sampled vines. Despite the usefulness of the most modern technologies (Figure 4), we still have a tough time sampling vines without the destructive effect. Therefore, new methods should address the maintenance of the sample analyzed. Hence, biosensors, resourcing to DNA hybridization, can further resolve some of the disadvantages associated with PCR-based methods, such as the need for smaller sampling, specialized handling, and expensive equipment, with the advantages of a fast response, cost-effectiveness, and portability for *in situ* measurements (Barrias et al., 2019). The use of specific biosensors can be an appropriate measure to help in the crusade against GTDs and even other vine-affecting diseases.

## Conclusion and future perspectives

The colonization of new areas by grapevine brought new and extensive sources of economic revenue due to the increased production of grapes and processed products, mostly wine. However, the lack of knowledge and the increased exportation of grapevine plants led to the widespread of several diseases, predominantly being GTDs. Since these pathogens can stay dormant, due to their endophytic lifestyle *in planta* for years, there is a pressing need for the development of fast detection methods that can be employed in the early stages in order to mitigate economic losses.

The main goal, so far, for vineyard protection and related economic aspects is the swift development of methods for precocious detection of GTD-involved inoculum capable of giving rise to outbreaks. Also, precocious detection of black foot and Petri disease pathogens in nurseries could diminish its propagation to fields. Several methods and protocols have been implemented to help mitigate and reduce the nefarious effects of GTDs, which range from controlled nursery practices during plant processes to field control using chemical agents, antagonistic microbiota, new methods of vine management, and disposal of the infected plants (Graniti et al., 2000; Agustí-Brisach and Armengol, 2013; Mondello et al., 2018). However, these are not fully effective, due to the ubiquitous character of the fungal pathogens, increased resistance to chemicals, and badly timed applications of these safety practices, leading to a partial reduction of the causal GTD agents and hence a less effective protection of the vineyards, as thoroughly explained by Gramaje et al. (2018).

Thus, it is mandatory that early pathogen detection in the vineyard and nurseries is achieved, and other possible inoculum sources are avoided in order to maintain grape yield and quality. Additionally, fungal detection in nurseries is also an urgent demand to be met, since undiagnosed vines pose another threat level to healthy vineyard implementation. The early pathogen detection and identification would allow the definition of manageable and focused actions against the targeted pathogen, thus enhancing mitigation measures. Also, molecular tools would allow to control the plant material exchange among countries and the transport of asymptomatic infected vines which could further avoid the human-mediated dispersion of pathogenic fungi to healthy vineyards (Travadon et al., 2012). Therefore, directives should also be updated to englobe potentially grapevine affecting fungal species, as is the case of grapevine trunk diseases.

Currently, the techniques available are limited, since they only allow a reactionary response, lack preventive measures, and do not act on early reduction of these pathogens, which remain “hidden” in the absence of external symptoms of a presumable healthy plant. Hence, early and fast detection of these pathogens is mandatory for integrated vineyard protection, allowing to protect the cultural and genetic patrimonies and economic interests of several grape-producing countries.

Detection of GTDs based on more classical approaches has proven to be insufficient to detect the actual problem, and hence methods for early detection of the pathogens should be widely implemented, which would allow more precise mitigation measures. Overall, fungal pathogen detection and identification *in planta* in the early stages of infection are more easily obtained by molecular-based technologies, namely, DNA detection. Furthermore, new insights are needed, since several aspects are not well-established for GTDs, such as the possibility of co-infection by other fungal species or adjuvants that may help manifest the disease faster or more aggressively (e.g., the pathogenic effect of *Seimatosporum vitifusiforme* when co-inoculated with *Diplodia seriata*, with *Seimatosporum luteosporum* in co-infections with *Diplodia ambigua* in vines of *Vitis vinifera* cv. Pinot Noir) (Lawrence et al., 2018) and the worldwide prevalence of the pathogens in distinct locations, since these diseases have a worldwide distribution with variation in the causal agents (Agustí-Brisach and Armengol, 2013).

Additionally, implementation of new technologies of metagenomic protocols will allow not only for pathogen detection and identification, thus helping in vineyard management, but also with the definition of GTD etiology by assessing microbiota in the vineyards and in the environment, hence unveiling new interactions between several microorganisms and new possible sources of biocontrol agents (Marraschi et al., 2019; Trotel-Aziz et al., 2019; Almeida et al., 2020; Niem et al., 2020; Russi et al., 2020; del Pilar Martinez-Diz et al., 2021; Cobos et al., 2022). Recently, mycovirus has been described as a causal agent of GTD; nevertheless, further studies are necessary to fully understand their role in symptom manifestation and their link to a specific GTD pathogen (Nerva et al., 2019). Also, implementation of mRNA analysis in pathogen–*Vitis* interactions, genome-wide sequencing, and metabolome-wide analysis of GTD pathogens enables the possibility of finding new answers related to dissemination, infection, and colonization pathways, hence helping to develop new alternative methods to tackle this problem (Cobos et al., 2010; Blanco-Ulate et al., 2013; Morales-Cruz et al., 2015; Paolinelli-Alfonso et al., 2016; Xing et al., 2019). Furthermore, methods that detect and identify the presence of pathogens, either by DNA amplification or DNA sensing, in vineyards before the appearance of symptoms would allow for more precise and early measures to diminish the pathogenicity of fungal inoculum (Pouzoulet et al., 2017; Billones-Baaijens et al., 2018; Dissanayake et al., 2018), thereafter complementing the actions needed to avoid extensive yield and vine losses.

## Author contributions

FA-N: writing original draft. PM-L: conceptualization, writing – review and editing, supervision, and funding acquisition. DG and CR: conceptualization and writing – review and editing. HG and AF: writing – review and editing. All authors contributed to the article and approved the submitted version.

## Supplementary material

Supplementary Information 1 Current knowledge of fungal species related to GTDs (Phillips, 1998; Fischer and Kassemeyer, 2003; van Niekerk et al., 2004; Halleen et al., 2007; Alaniz et al., 2009; Tomoiaga et al., 2009; Úrbez-Torres and Gubler, 2009; Trouillas and Gubler, 2010b; Luque et al., 2012; Agustí-Brisach and Armengol, 2013; Arzanlou et al., 2013a,b; Úrbez-Torres et al., 2013; Linaldeddu et al., 2014; Rolshausen et al., 2014; Arzanlou and Narmani, 2015; Cloete et al., 2015; Travadon et al., 2015; Maharachchikumbura et al., 2016; Carlucci et al., 2017; da Silva et al., 2017; Lawrence et al., 2017, 2018; Halleen et al., 2007; Pouzoulet et al., 2017; Váczy et al., 2017; Billones-Baaijens and Savocchia, 2018; Guarnaccia et al., 2018; Moyo et al., 2018, 2019; Pintos et al., 2018; Rezgui et al., 2018; Abed-Ashtiani et al., 2019; Akgül et al., 2019; Lesuthu et al., 2019; Manawasinghe et al., 2019; Raimondo et al., 2019; Amarloo et al., 2020; Berlanas et al., 2020; Brown et al., 2020; Lengyel et al., 2020; Mundy et al., 2020). Taxonomic synonyms of interest were obtained from Mycobank (2021) database (www.mycobank.org). Esca (Es), Petri disease (belonging to Esca complex) (PeD), Botryosphaeria dieback (BD), Eutypa dieback (ED), Phomopsis dieback (PD), black foot disease (BF), and Cytospora Canker (CC). Families/genera which are not recognized to be involved in Petri Disease, and therefore young vines, but have a role in mature vines affected by esca are indicated (Es^§^). Taxa which are considered to enhance GTD development as infections adjuvants (IA) are also indicated.

## Abbreviations

GTD: grapevine trunk disease
PCR: polymerase chain reaction
qPCR: quantitative PCR
ddPCR: digital droplet PCR
NGS: next-generation sequencing.
